# Nurturing Spiritual Resilience to Promote Post-disaster Community Recovery: The 2016 Alberta Wildfire in Canada

**DOI:** 10.3389/fpubh.2021.682558

**Published:** 2021-07-23

**Authors:** Nasreen Lalani, Julie L. Drolet, Caroline McDonald-Harker, Matthew R. G. Brown, Pamela Brett-MacLean, Vincent I.O. Agyapong, Andrew J. Greenshaw, Peter H. Silverstone

**Affiliations:** ^1^School of Nursing, Purdue University, West Lafayette, IN, United States; ^2^Faculty of Social Work, University of Calgary, Edmonton, AB, Canada; ^3^Department of Sociology & Anthropology, Mount Royal University, Calgary, AB, Canada; ^4^Department of Computing Science, University of Alberta, Edmonton, AB, Canada; ^5^Department of Psychiatry & Director, Arts & Humanities in Health & Medicine MD Program, and Faculty of Medicine & Dentistry, University of Alberta, Edmonton, AB, Canada; ^6^Department of Psychiatry, Faculty of Medicine, University of Alberta, Edmonton, AB, Canada; ^7^Department of Psychiatry, Faculty of Medicine & Dentistry, University of Alberta, Edmonton, AB, Canada; ^8^Department of Psychiatry, University of Alberta, Edmonton, AB, Canada

**Keywords:** spirituality, resilience, post disaster recovery, community, service providers, meaning making

## Abstract

The 2016 Alberta wildfire, the largest insured natural disaster in Canada, led to a mass evacuation of residents of Fort McMurray, a small city in northern Alberta. The wildfire resulted in significant damages to housing and community infrastructure. The entire community was displaced for several weeks. Post-disaster, community members experienced individual and collective trauma, and other negative mental health impacts in response to the significant losses and grief they endured. Spirituality has been found to be a major protective factor in facilitating resiliency and recovery following the experience of disaster. Nonetheless, little focus has been directed toward how spirituality can strengthen and empower community capacity and growth during post-disaster recovery. Our study explored various meanings and concerns, along with tools and strategies that helped to nurture spiritual resilience and well-being among residents of Fort McMurray following the Alberta wildfire. Data were collected through interviews and focus group discussions with community influencers working to support long-term recovery efforts in the city. Participants identified a number of spiritual resources such as a strong sense of belonging, a shared positive outlook, faith and hope, compassion, and sense of gratitude, which contributed to increased resilience and positive health and well-being and helped them to support families and communities in the post disaster recovery period. Our findings indicate that spiritual values and beliefs can play a significant role in building resilience and promoting individual and communal healing and recovery post-disaster. These findings have important implications for post-disaster recovery strategies, as they highlight the need to ensure supports for interventions and initiatives that strengthen a collective sense of identity and social cohesion, informed by communal norms and beliefs, including programs and resources which support opportunities for reflexivity to foster shared healing and ongoing recovery processes.

## Introduction

“*How we are able to weather the storm, how we're able to endure the challenges and complications of life that are thrown at us is a sign of our resilience. It's [pause] if we can't handle those, well then, we usually crumble and fall apart. But if we are still able to stand after experiencing trauma or tragedies like the wildfire and still stand on our feet that is a sign of resilience.” (Interview participant)*

The above quote describes a painful yet meaningful and self-enduring experience of an individual who experienced the Alberta wildfire disaster in 2016. The Alberta Wildfire which occurred in May 2016 is the costliest disaster and mass evacuation in Canadian history, resulting in damages totaling ~C$9.9 billion dollars (1). Over 88,000 residents were evacuated and displaced from Fort McMurray, Alberta, and ~2,600 homes and 589,995 hectares of land and other infrastructure were damaged (2). Shortly after the wildfire, the Wood Buffalo Ministerial Recovery Task Force was established to ensure safety and security in disaster affected areas, to support the community, to plan for the timely re-entry and settlement of residents, and to resume municipal, social, economic, and business activities (3). Voluntary re-entry was initiated in June 2016 in Fort McMurray, and the community rebuilding and recovery efforts are still ongoing today. The community as a whole experienced significant trauma, suffering, and difficulties as a result of this mass incident. 4 years later, many individuals in the community of Fort McMurray are still struggling to fully recover from the significant losses and prolonged distress that occurred as a result of the wildfire (4). In addition, other challenges which have occurred post-wildfire, including a prolonged economic downturn, particularly in the oil and gas industry which Fort McMurray is largely dependent on, a damaging flood in 2020, and more globally, the COVID-19 pandemic have compounded the challenges experienced in Fort McMurray and further complicate the recovery process. The recovery efforts are still going on in the community and various programs and activities are targeted to help the families and community in their post disaster recovery efforts. This article aims to discuss some of these enabling experiences along with spiritual tools and resources that helped community influencers and service providers to help families and community in post-disaster recovery and healing efforts.

### Fort McMurray Context

Multiple geographical, social, economic, and cultural influences shape the resilience and wellbeing of a community especially in the post disaster recovery context, therefore it is important to recognize the unique context of the Fort McMurray community. Fort McMurray is the urban service area that lies within the Regional Municipality of Wood Buffalo (RMWB) in Northeastern Alberta on Treaty 8 land and serves as the municipal center for several multi-nationally owned oilsands projects that surround it. Prior to the wildfire, the municipality was home to a total of 111,687 residents, of which two-thirds resided within Fort McMurray itself. It is a diverse community in that approximately one-third of the entire population is non-permanent/temporary resident, also referred to as the “shadow population.” These temporary residents include both regional and international mobile workers who are mostly employed by oil companies in the region. Ninety percent of these workers resides and works within the active work camps near oilsands projects sites (4). Dorow and O'Shaughnessy (5) describes FMM as a uniquely diverse community comprised of mobile workers, permanent residents and aboriginal communities having varied social, cultural, and economic ties with the place they live. As per the RMWB census report, 2018, there are more males 54.9% as compared to 45.1% female population. Due to steep housing costs and transient population, homeowners account for 63% of the population within Fort McMurray, Alberta (~10% lower than the provincial average). Prior to wildfire, in 2015, FMM also faced an economic downturn due to lowered oil prices, further worsened by the disastrous wildfire in May 2016 resulting in a mass evacuation, with physical infrastructure destroyed and other damages in the Town (4). As per the census reports from Regional Municipality Wood Buffalo (2015–2018), there has been a 10.67% decline in the total population and a 14.9% decline in the shadow population within these 3 years nonetheless the age, ethnicity, and gender structures remained more or less the same within the region. Nearly 19.3% of residents have relocated within the Municipality due to wildfire and other social and economic reasons (4).

### Spirituality, Resilience, and Post-disaster Recovery

Disaster impacts physical, economic, spiritual, and psychosocial well-being, inducing traumatic responses among those affected, particularly those who are most vulnerable, including the poor, children and youth, immigrants and refugees, and disenfranchised groups (6). Along with the trauma of experiencing a disaster, individuals and families often experience the loss of homes and community infrastructure, which makes the basic tasks of everyday life challenging, as well as loss of a sense of identity, meaning, and purpose—all of which impact overall health and well-being (7). Benson et al. (8) note that following a disaster event people often enter into helping relationships with feelings of helplessness, loss of personal control and doubt about their relationships, environment, and their cultural and belief systems. The struggle to find meaning in their losses and trauma can result in various mental, psychological, and spiritual issues, and delayed recovery. A complex process, post-disaster recovery requires a shared sense of solidarity that develops through collective expression of grief and empathy, communal commitment, and sense of public duty directed to uniting communities, building resilience, and fostering overall well-being (9). Shared values leading to emergence of social cohesion motivates and enhances the ability of the local population to adapt, respond, and cope in the aftermath of disaster (10).

Ungar (11) has described resilience as a set interdependent processes that “reflect the positive adaptations that individuals, families, and communities make” (p. 255) following exposure to trauma. He characterizes resilience as the co-occurring capacity of individuals to navigate their way to the psychological, social, cultural, spiritual, and physical resources that sustain their well-being, and individual and collective capacity to create or obtain culturally meaningful resources needed to support such efforts (11). More specifically, spiritual resiliency is defined as the ability to sustain one's sense of self and purpose through a set of beliefs, principles, or values while encountering adversity, stress, and trauma by using internal and external spiritual resources (12). The literature indicates a positive relationship between resilience and spirituality both at the individual and the community level.

Spirtuality is defined as an aspect of humanity that refers to the way individuals seek and express meaning and purpose, the way they experience their connectedness to the moment, to self, to others, to nature and to the significant or sacred (13). Individuals often identify as being spiritual, regardless of whether they are religious or not. Wattis et al. (14) asserts that spirituality can be viewed both in secular and religious terms and is no longer confined to religion. Most authors now view spirituality and religion as separate and distinct constructs whereas some may them as overlapping concepts (15). Spirtuality can be expressed in religious and non-religious forms and values, some people express their spirituality through their religion such as prayers and meditation; others may express through non-religious forms such as showing love, being present, community participation, listening, and communication in forms of art, drama, and music etc. (16). Individuals often seek spirituality or find spiritual resources when suffering as it helps them to find meaning, endure hardships and adversities, and supports healing and recovery during periods of loss and grief; experiencing a disaster and the associated recovery processes post-disaster (17–19). Spiritual needs or resources may include need to find meaning, love, sense of belonging, hope, peace, and gratitude (20).

Given this, it is clear that spirituality can be an important aspect of resiliency as it contributes to adaptive coping as well as personal growth and transformation following exposure to traumatic events and stressors, including disasters (10). Spirtuality strengthens family relationships and promotes healing and resilience through coping, inner peace, self-esteem, perseverance, and helping others (20). Additionally, it assists people in adapting and transitioning to, or constructing a new normal following disaster by providing a positive worldview, meaning and purpose, psychological integration, hope and motivation, personal empowerment, a sense of control, answers to ultimate questions, or meaning, and social support (21). Despite the significant role that spirituality plays in resiliency processes, limited research has been directed toward identifying the various means, approaches, and tools related to spiritual resilience which help strengthen and empower community capacity and growth during post-disaster recovery.

According to Walsh (20), spiritual values and beliefs influence the ways individuals and families deal with adversity and suffering, through the meanings they associate with these experiences. Walsh (20) outlines how these values influence the ways individuals and families communicate about their pain and struggles, as well as their attitudes toward mental health and health care, and their preferred pathways to recovery. Spiritual values and resources—such as meaning making, hope, self-efficacy, strong sense of community identity, relationship building, and belongingness—have been found to be powerful resources in fostering the resilience of communities. These values and resources serve as important protective factors in helping individuals cope with unimaginable losses, resulting in reduced impact on mental and psychological health (10, 21). Thus, it is important to address spirituality as a means of making meaning of, and recovering from, adverse events like disasters, and explore ways to build community resilience through this dimension as part of post-disaster recovery processes.

Disasters often pose threats to individual's meaning making experiences, as they can remind individuals of their mortality, threaten the predictability and safety of the natural world, and cause existential anxiety leading individuals to question the larger meaning of life (22). Park's meaning making model has been increasingly used as a framework to conceptualize resilience and coping abilities among individuals and communities, particularly in the context of disaster and the disaster recovery process (10). Park states that survivors' ability to engage in, and approach meaning making is greatly influenced by their religious and spiritual values and beliefs. In the aftermath of disaster, families often experience a strong sense of helplessness and lack of security due to major threats to their physical and psychological resources. These experiences often result in feelings of meaninglessness and lack of purpose, resulting in mental health challenges such as depression, and psychological and spiritual distress. Building on Park's (10) meaning making model, how individuals understand, interpret, and reappraise the guiding meanings that direct their lives, and plan to adjust their life goals and actions, influences their resiliency, overall recovery process, and future outcomes. Understanding those meanings, behaviors, and actions can guide professionals in planning appropriate community interventions to foster community resilience in the post-disaster recovery phase.

Multiple studies have found that spiritual meanings and resources have shown positive impacts on post-disaster recovery outcomes among families and communities. Haynes et al. (22) found that spiritual meaning and sense of peace buffered the deleterious relationship between resource loss and symptoms of spiritual distress among survivors of Hurricane Katrina. Survivors who reported experiencing higher spiritual meaning following the disaster reported significantly less severe post-traumatic stress in response to resource loss, relative to survivors who reported lower spiritual meaning and peace. Similarly, Alawiyah (21) found that spirituality and religious practices provided the motivational force that supported resilience among African American survivors of Hurricane Katrina. Other studies have also reported that spirituality was an important resource that contributed to enhanced community resilience, and also helped to reduce post-traumatic mental health impacts and promote healing, through improved social support and enhanced self-efficacy during post-disaster recovery periods (23–25).

### Study Context

The findings of the following analyses draw from a larger study on the “Health Effects of the 2016 Alberta Wildfire: Pediatric Resilience” that primarily focused on examining the effects of the Alberta wildfires on children, youth, and families to better understand the social, economic, spiritual, and cultural factors that contributed to overall well-being and resilience. Individual characteristics and social-environmental factors in the lives of children and youth were considered in the recovery context to gain a holistic understanding of their health, functioning, and overall well-being. Brown et al.'s (26) population survey in a similar context showed that there was a significant negative impact of the wildfire disaster on many aspects of adolescent (grade 7–12) mental health and a significant increase in symptoms related to depression and suicidal thinking. To gain further insight into community resilience and the impacts of disaster, qualitative interviews and focus groups were conducted with community influencers in Fort McMurray. “Community influencers” is a term used in Alberta, Canada, that refers to direct service providers, community leaders, social work practitioners, educators, and individuals delivering services and programs to children, youth and families in a variety of organizations (6, 27). Drawing from the collective narratives gathered from community influencers, this article discusses the role of spirituality and spiritual values in fostering community resilience during the post-disaster recovery period.

## Methods

A qualitative descriptive study design was adopted using a community-based research approach. A research partnership was created with the Public and Catholic School Boards of Fort McMurray, Alberta, and other local community partners for the study (4). Qualitative interviews and focus groups were used to gather the perspectives and experiences of community influencers engaged in the delivery of services and programs for children, youth, and families, post-wildfire. Using a purposive, snowball sampling approach, participants were recruited from: (1) social service agencies and community organizations based in Edmonton and Calgary (the other two largest cities in Alberta) which played an important role in addressing the immediate needs of Fort McMurray residents following evacuation (housing, food, clothing, wildfire response updates, mental health support, etc.), and later supported their return home; and (2) social service agencies and community organizations based in Fort McMurray that actively supported the re-entry of residents, and have continued to support ongoing recovery efforts. These social services agencies and community organizations include but not limited to municipalities and regional services, schools, faith based organizations, recreation/wellness centers, child and youth crisis support services, culturally focused services, and mental health support services (For sample demographic information, see Table 1) For recruitment, phone calls and separate email invitations were sent to the agencies along with the study information letters by the principal investigator (PI) of the larger grant study with the post-doctoral fellow and four research assistants who were also part of the study. Semi-structured individual in-depth interviews were conducted by both PI and research fellow in the study whereas all the focus group discussions were held by the PI along with a note taker in the study (4). The interview transcriptions were done by the research assistants and then analyzed by the research fellow along with the PI and Co-I of the study.

**Table 1:** Demographic information for interview participants in Fort McMurray.

**Community Agencies/Service Providers**	**# of Participants**
Community Schools (Public/Catholic)	10
Non-Governmental Organizations (NGOs)	4
Wood Buffalo Municipality District Offices	5
Youth Elder (Aboriginal Organization)	1
Faith Ministries (Church Services)	2
Mental health/Wellness Services/YMCA	6
Alberta Health Services	2
Total	30

All the individual interviews and focus groups were held in person. Thirty interviews and 12 focus groups were conducted, yielding a sample of *n* = 30 and *n* = 35 participants, respectively. The small number of participants in each focus group session allowed for a rich and descriptive discussion of key questions and issues. The average duration for interviews and focus groups was 1–2 h. Interviews and focus group discussions focused on examining the perspectives and experiences of community influencers with respect to their roles in supporting children, youth, and families during the wildfire evacuation, response, and re-entry and recovery stages post-disaster. Focus groups were conducted in person with the objective of presenting preliminary interview findings to the participants and eliciting their feedback on identified themes, and further exploring their perspectives about the challenges the community faced, and how these could be addressed more appropriately. Research ethics approval was obtained from the Ethics Review Board at the University of Calgary. Participants' written consent was obtained prior to data collection; all data collected were audio-recorded and transcribed for analysis by the research team.

Data analysis was informed by the interpretive constructivist paradigm, recognizing the multiple, shared socially constructed realities of people's lived experiences (28). Using constructivist grounded theory concepts (29, 30), this paradigm was used to identify and explore multiple, subjective perspectives regarding ways spirituality can strengthen and empower community capacity and growth during post-disaster recovery processes. Grounded theory moves beyond individual perspectives to understand the meanings that groups of individuals attach to their experiences and the world around them, capturing patterns revealed across individuals and groups, including those that might not be obvious to participants (31). The emergent, inductive, and comparative nature of Grounded Theory research rendered it particularly well-suited for analysis of the qualitative data that was collected in this study. Individual in-depth interviews and focus group discussions were transcribed for coding and analysis by the research team. Separate memos and contextual details gathered during the interviews and discussions were also added to the data. NVivo 12.0 was used to support qualitative data analysis of the transcripts. The PI and Co-I along with the graduate research assistants analyzed transcripts to identify emerging themes, links, and associations embedded in the data. Coded quotes were extracted for preliminary analysis, compared within and between cases, and grouped according to key themes and sub-themes. To maintain validity and reliability, a penultimate thematic listing was shared among research team members for review. Following minor clarifications, the final thematic listing was determined based on consensus.

## Findings

Analysis of data gathered from the diverse group of community influencers who participated in individual interviews and focus group discussions provided a vivid description of their experience of the 2016 wildfire, the chaotic city-wide evacuation, response and early and ongoing recovery processes since residents have returned to Fort McMurray. The narratives shared by the participants demonstrate several meaning making and spiritual perspectives, inner resources, and tools that helped families and communities in their post disaster recovery and healing processes. It is significant to note that participants were not asked directly to identify themselves as spiritual or not spiritual during the interviews or focus groups, nonetheless the narratives provided by the participants and extracted themes indicated various spiritual perspectives, attributes and inner resources that fostered resiliency and helped community to cope and adapt to the new normal in the post disaster recovery and healing process. These perspectives were organized into several themes under the study. The major themes included: a strong sense of belonging and social connection, fostering a shared positive outlook, hope and faith, sense of gratitude, compassion, and altruistic values, as well as strategies and programs used to foster spiritual resiliency among different groups.

### Strong Sense of Belonging and Social Connection

Participants highlighted a strong sense of belonging and relational connectedness that Fort McMurray residents shared, which helped them to work collectively toward recovery and healing efforts. Although, participants also shared that they still feel a vacuum for those residents could never come back to the community due to various social and economic reasons and wished that they would never have to lose those connections. Most participants view the community connectedness and togetherness as a strength and an important resource to motivate and boost their efforts in post-disaster recovery period. Participants described the importance of maintaining and strengthening a sense of community post-disaster by reinforcing social bonds and continuing to grow together as a community. One focus group participant shared:

*Fort McMurray is so much more than the fire. I think the outside community thinks it's the place of the fire. Although it's a big part of our story it's not who we are. I encourage all people that once we get to a place of help, that this is an opportunity for us as a community to grow our character. We would be stronger, more loving, caring, and noticeable of the world around us. (Focus group participant)*

The above finding was surprising yet significant in the study knowing that Fort Mc Murray is quite a diverse community comprised of temporary residents/mobile workers which make up one third of the community. Despite this unique aspect of the community, the participants expressed values of strong social ties, connections, and respect for each other. Such a unique population demographic of Fort McMurray was identified as a key consideration by participants in the study, which is evident in the following statement by one of the focus group participants:

*The other strength is that because you had people that came from all around Canada and the world to live there [in Fort McMurray], they have some very strong bonds … that aren't necessarily the traditional bonds that we see with our own families and extended families. (Focus group participant)*

Many service providers agreed that community connections were disrupted by the wildfire during the evacuation and it was important to re-establish those connections and relationships after the wildfire. For example, one focus group participant stated:

*But the bigger picture was connecting, because after the evacuation everybody felt like we were isolated in our own little silos, because we were all going through different (things), we were all impacted differently but, we were nonetheless impacted in what happened…. We are a relational community here. We publicly teach the importance of community and relationship all the time. Other than being in close relationship with other people, it's hard to support and help people. (Interview participant)*

Most of the participants discussed the wildfire recovery in relation to strengthening social connections and social capital in the community. This is evident in the following statement shared by a focus group participant:

*[Recovery] is really about those social connections and building that social capital (Focus group participant)*

Participants expressed that a successful post-disaster recovery meant moving forward together, understanding and respecting the needs and concerns of affected individuals and families, and ensuring a strong sense of community by sharing love and care, providing timely and adequate support, and pursuing communal growth. They strongly believed that it was necessary to help each other build their individual and collective capacity for realizing the future promise and potential of the community. Individual failure was viewed as a community failure. Participants emphasized the need to care for, and support everyone on their respective journeys through loss, grief, and trauma as they moved forward toward healing and recovery. As one interview participant stated:

*Success to us is, are people coming together to move people along in their journey. For us, real success is often deemed by are we being an encouraging family to one another. Are we loving one another? To the point of seeing others move and grow. Growth can look different for different people and be at different paces for different people, but if somebody can't get to the next step because they don't feel love or supported; that's a failure to me. Not every person can meet every goal, not everyone can be the Prime Minister. That would be awful if we had 32 million Prime Ministers, right? Not every person is going to reach the same end goal, but if every person is not able to reach the potential in them, then we failed as a community. (Interview participant)*

Community connections were also perceived by participants as an important element of fostering a sense of belonging, particularly among immigrant newcomers. One of the focus group participants stated:

*We deal with families that are newcomers to Canada and they have no idea what they are going through, how to deal with it and where to go, and that is very important for us to give the family a sense of belonging and that would help them a lot, you know … so it depends about who they are and where they come from because this is a multicultural society, so something, having a sense of belonging, guiding them to where people speak their own language so they felt they belong, that helps them really significantly with these situations. (Focus group participant)*

Participants also discussed how mental health is strengthened through social and community connections.

*Mental health is not created in isolation; it is created through connections. (Focus group participant)*

Events such as community dinners were purposefully organized by community organizations and service providers to foster relationship building and a sense of belonging among community members. According to one interview participant, such events helped to promote a sense of togetherness, that generated a sense of cohesion and unity.

*We did community dinners which brought people in the community together, and they didn't have to talk about the evacuation, the fire if they didn't want to, it was just for the dinner to sit. And we would go into different communities who were mostly impacted by the fire and would sit with them. Sometimes we would show them like a little film, and talk about mental health. But sometimes it was just about offering support, listening to music and talking, and socializing. (Interview participant)*

In discussing post-wildfire resilience, some participants discussed the importance of keeping the needs of children and youth in view. Participants recognized the importance of children having a stable adult in their lives to provide support and reassurance and be a positive guiding influence. This is evident in the following quote from a focus group participant.

*I think relationship building is probably the number one thing we have witnessed. We have some children that just really need an adult in their corner, a stable adult in their corner. (Focus group participant)*

In addition to relational connections and mutual support, participants also mentioned strategies that fostered a shared positive outlook.

### Need to Foster a Shared Positive Outlook

Study participants identified that a shared positive outlook was integral to helping community members cope with the impact of the wildfire. While this was recognized as important, participants shared that maintaining a positive outlook was a challenge for many community members given the scale and devastating impact of the wildfire. Participants shared that most families are still experiencing post-traumatic and anxiety symptoms along with other mental health issues and were finding it challenging to cope with multiple psychosocial and other stressors caused by the disaster. In most instances, mental health was not given an immediate priority among other various physical needs such as loss of homes, clothing, food, insurance, and loss of jobs. According to one focus group participant:

*This is an entire community that is facing PTSD, and facing trauma, and facing a lot of challenges. (Focus group participant)*

Participants also noted multiple associated, intersecting stresses and pressures, such as waiting for reconstruction of homes, coping with other physical and social losses, and loss of employment and income associated with the ongoing economic downturn. In addition to making it difficult to maintain an optimistic outlook, these challenges negatively impacted the mental health and spiritual well-being of community members. As one of participant shared:

*The challenges have been multifaceted because not only is it the fire, it is the slowdown, the layoffs, the change in hours at work, so families that were well-to-do are maybe just trying to get by on one pay cheque. (Focus group participant)*

Many participants also explained that disaster-affected residents were focused on meeting their basic needs, and the need for mental health support emerged later in the long-term recovery process.

*It is hard to focus on your mental health when you are in survival mode and you are trying to meet your basic needs. (Focus group participant)*

Another interview participant pointed to the complexity and challenge of fostering a shared positive attitude, given the unique experiences and array of challenges facing everyone:

…* My story is that 88,000 people left that day, and there's 88,000 stories, and 88,000 ways people were impacted. Even for myself, my husband started smoking again, and I put on 20 pounds. Everybody has a different way of coping and trying to find ways to recover from that is difficult. Once you're in that, it's hard to pull yourself out. As time goes on, you just kind of throw your hands up and say OK, I'm done. (Interview participant)*

Service providers strongly advocated for the need to draw on the inner strength of individuals that could contribute to empowering the collective effort of community members in rising to address ongoing challenges and working toward creating a more positive future. One service provider shared:

*Despite multiple challenges, you can find the inner strength to pick yourself up and move on. Knowing that when you move on it may not look like what it looked like in the past, but you can still make positive choices for yourself. You don't have to be defined by the event that happened*. … *Life continues to present some challenges, but hopefully you can give people the tools and skills to actually learn forward and cope better. (Interview participant)*

In the midst of the above challenges, hope and faith emerged as major resources for building spiritual resiliency in the post-disaster context.

### Hope and Faith

Participants in the focus group discussions reported that instilling hope and faith was expressed as “believing that something good is coming along the way.” Faith and hope emerged as major resources in the provision of a collective sense of empowerment and growth, as well as courage to cope with all the losses and hardships experienced post-disaster. Community dinners and other congressional activities were held monthly in community spaces such as schools and playgrounds. These programs created space for community members to share their experiences, discuss their grievances, express their emotions and feelings, and build hope for a better future. This is evident in the following statement by one of the interview participants:

*Our resiliency is centered on hope. In a sense that, in order to be resilient, we need to know that there's hope for better and something different. The hope comes through our faith. We believe that God can bring us to better ends and has good plans for us. It's difficult to be resilient if you don't think there's a good place to get to. Why put in all this effort, get out of bed and fight through for all these things, to end up in somewhere you don't want to be. For us, a lot of it is reminding and teaching people there's always hope. The importance of being the voice in each other's life, of being hope, when someone can't see it themselves. (Interview participant)*

Participants working with children and youth in elementary and secondary schools highlighted the importance of hope in their work. One interview participant shared:

*What I want to see is students thrive, have hope, and believe that we can have a better life before. That's where I'm trying to get … The core of our being, our faith, is having hope that we can actually do better. (Interview participant)*.

Some participants identified churches as a spiritual place where community members could meet.

*You need to go to your church and hang out with a friend, to go for coffee. (Focus group participant)*

Similarly, hope was also expressed as pride in the community's rebuilding efforts post-wildfire. A focus group participant explained:

*It's a kind of community pride that I think a lot of youth had, especially the ones who came back, they love their community. And now, it seems maybe in their mind something else is now threatening their community, that they have worked so hard to recover from the fire. (Focus group participant)*

Instilling hope through community rebuilding, as well as other opportunities for gathering together to discuss their fears and anxieties, along with their hopes, commitment, and resolve, and sharing their faith practices, helped to foster a positive and supportive environment that facilitated post-disaster recovery for many in the community.

### Sense of Gratitude

Participants explained that despite needing to grieve and adjust to difficult losses, the major strength of the community was found in the gratitude they experienced, the connections they built and the inner courage shown to help themselves and others in the midst of several challenges and adversities. One interview participant stated:

*We need to acknowledge that our life isn't just about possessions, it should be about community, love, life, hope, and joy. All the things that can't be measured by what's lost in a fire… Be thankful to the Creator for giving us the day, look positively, and try to carry on with your work. At the end of the day, you'll be happy. (Interview participant)*

Many participants expressed a sense of gratitude to people across Alberta, and the entire country, for all they did to support wildfire recovery and rebuilding efforts in the community.

*I think Albertans worked well. I think they were generous; they were conscious of what was happening, and there is that ethos in most of our communities that says ‘We will help. Let us know how to help and we will help'. (Focus group participant)*

One participant described how the generosity of people from across Canada helped to instill hope and foster a positive outlook among community members.

*But, there's a whole lot of generosity within our whole country and helping our community get back on its feet with welcoming 80,000 people into households into—so I think it's really, I think the community has a lot of hope in it. Like, I think people feel pretty good about (it), even if they're going through a hard time, they're wanting to keep working toward it. (Interview participant)*

In addition to the sense of gratitude, community members were willing to help each other to develop a stronger and resilient community.

### Compassion and Altruistic Values

Compassion and being of service to others were also identified as inherent values held within the community which were viewed as essential in “building back better” as part of Fort McMurray's post-wildfire recovery. Participants felt a sense of responsibility to serve their communities and found joy in helping each other. One participant shared that despite her house being completely destroyed by the fire, she felt compelled to return to support the rebuilding of schools in the community. She explained:

*I couldn't move away or walk away from it. We were in crisis. I felt a significant responsibility to make sure things were put back in place. My biggest motivation is just working with the families, the children, the positives, the negatives, the challenges, the successes. It's … no day is ever the same … I really look forward to being able to help shape the lives of those little children and help them grow. (Interview participant)*

Altruism was expressed by participants as a concern for the well-being of others in the community who they felt closely connected through a sense of kinship given a shared attachment to Fort McMurray as being their “home.”

*This is my home. My family is here. My son is here. This is where I live. This is where I want to be. I think it's important that we take care of each other. I think it's important that everybody has a role to play in it. I feel like this is my place to help. (Interview participant)*

The development of relational connections through altruistic and compassionate commitment to helping others led to a sense of self-efficacy and positive future orientation. As one participant shared:

…* And I mean, with families you build those positive relationships, it's such a small community that everybody kind of knows everybody, and I can't see me doing anything else, you know! It's just, I love the people… If families did need support then it was having communication with the families, asking if they need help with anything, and being there to listen to them. It made us a lot closer with the family that we helped support. It was something we all went through together. It was a learning process when we came back. (Interview participant)*

Another participant described the remarkable efforts and practices that took place after the wildfire, and expressed a sense of pride which suggested a positive future outlook.

*It's good to promote the positive stories that happen when disasters happen. (Focus group participant)*

The development of a greater sense of self-efficacy, inspired by compassion, and motivation to be of service to others and the community, was described by many participants as a milestone in the post-disaster context.

### Strategies and Programs Used to Foster Resiliency

Several community-based, trauma-informed programs and strategies were implemented by community-based organizations to support post-disaster recovery in Fort McMurray. Efforts were made to engage new, often unanticipated individuals and groups in taking leadership to initiate resilience efforts. This stimulated innovative approaches to integrating resilience into different aspects of the community, and led to recognition and appreciation of strengths-based approaches to post-disaster recovery. One focus group participant stated:

*We certainly heard from community organizations around really sort of focusing more on a strengths-based approach. (Focus group participant)*

Designed for service providers as well as community members across all age groups, including children, youth, and families, these programs focused on improving mental, psychosocial, and spiritual health, and enhancing overall individual well-being. Participants highlighted several examples of initiatives that were introduced to support community members, which are described below.

#### Wellness Grant

The “Wellness Grant” enabled service providers to access funding for specific initiatives that aimed to help them cope with trauma and enhance their sense of self-efficacy, while providing support to individuals and families in the community. Several participants discussed how this program helped them cope, and maintain a sense of self-efficacy, post-disaster period. One participant shared:

…* The ‘Wellness Grant' … allows staff to do things like, coffee break. Where they bring in Tim Hortons coffee and muffins and donuts and everyone kind of gets to have a minute to sit, chat, catch up, and be together. So, it's not just an entire day of hustle and bustle, and running around, and making sure all the kids are okay, but making sure they're okay. (Interview Participant)*

#### Journey of Hope

The “Journey of Hope” program was developed to (1) support families experiencing complex challenges, such as grief and loss associated with separation and divorce, and domestic violence, that made reintegration into the community difficult; and (2) support children who were severely traumatized by the wildfire. The program aimed at helping vulnerable members of the community overcome fear and anxiety due to family disruption, and also those experiencing a heightened sense of anxiety and/ or isolation, post-disaster. The following quote by a participant speaks to the efficacy of the program:

*We have a partnership with a non-profit agency who runs a separate program for family separation, (and) divorce groups. Their providers also come into our school and do grief and loss groups as well. So any time and grief and loss is very, very you know it could be loss of a house, loss of a park, loss of a friend who's moved to a different community because of the fire. Their expert providers would come in and do grief and loss work with our kids as well as work with our families. (Interview Participant)*

#### Peer Mentorship

Peer mentorship and other mental health counseling programs were offered to children and youth in elementary and secondary school settings to help them cope with the trauma they experienced and promote positive socialization and community integration post-wildfire.

*I think our ‘Friendly Peer' program which is the substance prevention program would be huge. A lot of …, especially teens, when teens hit like Grade 7, and that's what the program is for. We can do it with Grade 6, but it's for Grade 6 up to Grade 12. I think talking about substances, turning [to] them when you're stressed out, or peer pressure, those types of topics. We spend about 3 to 4 weeks on stress, what stress is, and how to deal with it. It's okay to feel stress. Stress is never going to go away in your life. Especially when you get old as an adult, but here's a healthy way to deal with it, and why you shouldn't turn to drinking and those types of things. I love that program. It's a really great program for Grade 7 when they hit that age. (Interview Participant)*

#### Mindfulness and Psycho-Education

Mindfulness and psycho-educational programs introduced in the community focused on emotional regulation to facilitate the psychological, emotional, and social well-being of children, youth, and families affected by the wildfire. The following quotes attest to the value the participants placed on these programs. This participant endorsed mindfulness as an important approach for promoting healing and recovery:

*I feel that's a huge strategy, anxiety is a huge piece to work on because, whether it be in children, or in adults, we all suffer from anxiety to some degree, and having that psycho-education, and learning some of those strategies, those coping strategies of grounding, and you know, learning about ways of regulating yourself, and how connecting it to your body, and understanding what's happening within your body is helpful (Interview Participant)*

Participants also recognized how important it was to provide reassurance and support to families and children, especially when parents were still struggling with their own trauma post-disaster. One participant shared:

*We have ‘Circle Security', so that's working with families, and that's helping them understand the attachment piece, cause, often times, when you are working with families who have been traumatized, they forget, or they …, sometimes, it goes on the back burner how important attachment is with children, and … they're detaching because they are working through their own trauma, and they forget about how it's impacting their children. So ‘Circle Security' helps them understand, it gives them psycho-education about attachment, and then gives them strategies to work with their children, and their teenagers. (Interview Participant)*

Participants also pointed to the benefit of introducing specific emotional regulation approaches to students as a means of reaching their parents. One participant shared:

*This year we're doing ‘Heart Math', which is deep breathing and using a heart monitor to help regulate themselves. … We had the students teach the parents how to use ‘Heart Math'. One of the things we've learnt with this process is, it's very difficult to help the parents. They don't want help. They won't show up for sessions, but if we teach the kids, the kids will teach their parents. It's an effective way to impact them in a positive way. That would be true with ‘Leader in Me' too. For ‘Leader in Me', we trained all our teachers to look at their own ‘7 habits', like to ‘be proactive', and ‘begin with the end in mind'. That was the first step, now it's about how do we teach students to be leaders. Teachers support them in their leadership, and that seems to be working. (Interview participant)*

Overall, recognizing the diversity of needs, participants pointed to the need for a continuum of mental health support at the community level as part of an overall strategy directed to promoting resilience and enhancing well-being:

*There needs to be a whole range of supports … we have heard about peer support work … there just needs to be a whole range to meet people where they are at because of the uniqueness of people's needs. (Focus group participant)*

Participants shared that individual and collective efforts that had been committed to rebuilding the community–through these programs and other initiatives–had provided important opportunities for learning and growing, and enhancing collective resiliency by building upon each other's strengths, which had contributed to supporting the resilience, well-being, and long-term recovery of the community.

## Discussion

The challenges experienced by service providers and community influencers, as well as the community efforts that were implemented to rebuild the community during the post-disaster recovery phases were perceived by participants as an opportunity to learn, grow and build upon each other's strengths and experiences, thus increasing resilience. Participants identified a number of spiritual resources which contributed to enhancing resilience and promoting health and well-being post-wildfire, such as a strong sense of belonging, a need to demonstrate a shared positive outlook, faith and hope, compassion, and a sense of gratitude. The wildfire resulted in significant physical, psychological, and spiritual distress among families, children, and youth in the community.

Findings suggest that the strengths-based, spiritually informed programs and practices served as both protective factors and interventions that helped children, youth, and families cope with the challenges faced following the Alberta wildfire. This likely helped prevent ongoing spiritual and psychological distress, build resiliency, and facilitate a path toward long-term recovery. Community members were displaced for several weeks following the chaotic mass evacuation, and upon their return to Fort McMurray, they continued to be impacted by ongoing social and economic crises. Some residents left and were unable to return back to the community due to the socio-economic and other crises related to the disaster. Despite several challenges, the community strived to be united and demonstrate faith, compassion, hope and a strong sense of altruism and service to others. Building on their collective sense, meaning-making, and continued persistence and work, the community established and offered various services and programs to support and sustain recovery and resilience post-disaster. A range of spiritually relevant values and strong sense of community enabled residents of Fort McMurray to endure and make meaning out of the ongoing painful realities and hardships that often result from disaster. For some of those who believed in facing their fears together found increased ability to restore the strength and determination working together for the community recovery and healing efforts. O'Grady et al. (32) support a similar view that multiple traumatic life events including disasters result in an existential quest, and a broadened view of life, leading to an increase in spirituality. Our findings also support Park's (10) meaning making model, in that adverse events enhance the individual's or community's ability to develop positive meanings out of tragedy and help build a sense of inner strength and purpose in life, leading to potential growth and transformation. The spiritual resources and values identified in this study enhanced the community's ability to cope with loss and grief, and generated a sense of self-efficacy, which are major pillars supporting post-disaster resiliency and recovery (17, 33).

Post-disaster resilience and recovery is a long-term process, not the outcome of a specific program or policy. The study findings suggested a strong sense of social connectedness, unity, and shared values contributing to enhanced support and resilience among wildfire-affected community members, further amplified by a wide range of community-based strategies and approaches. Considering FMM as a unique diverse and mobile community, the demonstrated shared values and strong social cohesion carries a considerable significance in the study. Social relations and community interactions play a meaningful role in promoting coping and recovery, often resulting from the supportive efforts of friends, family, and community agencies, as well as a sense of community connectedness and care (24, 34, 35). Multiple authors indicate the importance of social cohesion, community governance, and communal identities in promoting collective efficacy and resilience as helpful in addressing collective trauma post-disaster (25, 34). Further, collective efforts and social connectedness increase opportunities for knowledge and resource sharing, and provision of improved social support mechanisms within the disaster-affected communities (25, 34, 36).

During periods of any disaster or crisis people frequently seek spirituality or spiritual resources in order to find meaning, sense of belongingness, find love, hope, compassion, a sense of gratitude and peace to support healing and recovery (10). These spiritual values and resources have all been associated with increased resiliency and improved mental and psychological outcomes in crisis and disaster situations. Haynes et al. (22) examined whether spiritual meaning can buffer the effect of disaster-related resource loss on posttraumatic stress among Hurricane Katrina survivors (*N* = 485). Their study found that the survivors experiencing higher spiritual meaning following the disaster reported significantly less severe posttraumatic stress as compared to survivors who reported lower spiritual meaning and peace. Similar other studies also found that people who exhibited high spiritual values and resources such as meaning making, finding connections, hope, and gratitude demonstrated a stronger adaptive coping and resilience (21, 23). Glass et al. (23) in their study found hope to be a critical spiritual resource that enhanced coping mechanisms and minimized general psychological distress among disaster-affected families and communities. Similarly, Kulig et al. (37) in their study examining post-traumatic stress among with young children post wildfire disaster also found that both internal and external protective factors such as self-regulation, encouraging supportive and caring relationships, fostering values of cooperation, positivity, and sense of security can buffer, ameliorate, and mitigate the effects of trauma and stress and thus, bring positive behavioral outcomes and enhanced coping. Similarly, our findings also indicated that programs like “Journey of Hope,” peer mentoring, mindfulness, and other psycho-educational approaches offered supportive opportunities for reflexivity that helped individuals and families make sense of and process their experiences post-disaster. This helped many community members develop sense of belongingness, find spiritual meaning, hope for the future during the post-disaster recovery process. Programs and approaches provided a sense of connection, as well as opportunities for shared meaning-making, both of which promoted a sense of security that helped engender self-efficacy and a more positive outlook. Additionally, psycho-spiritual transformation and collective growth and healing in the community post-wildfire helped build and strengthen resilience. The findings point to the importance of recognizing that those supporting community resiliency and recovery efforts, such as community influencers, can only do so if they themselves are supported.

Moreover, since the start of the COVID-19 pandemic, various virtual tools have been found useful to maintain and enhance individual's spirituality and build community resiliency (38). For example, video conferencing was used to connect family and community members when physical distancing was mandated by national or regional lockdown measures post disaster. Facilitated online group programs were used by community members as well as text message programs such as Text4Mood and Text4Hope Coronavirus Disease 2019 Pandemic: Health System and Community Response to a Text Message (Text4Hope) had been used to quickly distribute information on spirituality and resiliency as well as provide psychological support to residents affected by disasters (38).

In summary, findings from this study suggest that spirituality and spiritual values are integral in fostering resiliency among families and communities in a long-term post-disaster recovery context. Spiritual values and resources such as meaning making and belongingness, a deeper sense of gratitude, compassion and altruism were reflected in the collective actions of the community and were found to be integral to building spiritual resiliency among the disaster-affected communities. These spiritual resources generated hope, a sense of collective efficacy, empowerment, social cohesion, and resulted in psychosocial recovery, personal and social transformation, and growth at a communal level. Potential future studies are needed to generate further empirical evidence in understanding the role and relationship between spirituality, post traumatic growth, and collective resilience specifically in a disaster context. The unique demographics of FMM community raises several questions and insights for the researchers in understanding the unique sense of belongingness, bonding and commitment found among the community members, and could be further examined and studied. Future quantitative studies are recommended to examine associations among spirituality/meaning making, psychosocial recovery, and resilience promoting healing and recovery in post disaster related settings. There were also future calls for expanding programs like mentorship, counseling, psychoeducation and mindfulness to improve the spiritual resiliency among the families and community members. Funding for these programs should be considered. More studies can be conducted to examine the feasibility and effectiveness of these programs in improving spiritual resiliency and post disaster recovery and healing are also recommended.

## Study Limitations

Findings generated in the study are limited to the Fort McMurray area post wildfire.

A detailed audit trail was kept ensuring rigor, credibility, and trust worthiness in the study. There is of a possibility of socially desirable responses from the participants. The study did not ask directly from the participants if they identify themselves as spiritual or not and therefore, there is also a possibility of differences in spiritual perspectives among participants who identify themselves as spiritual or those who did not. The rich findings generated provide lessons and insights to consider and develop programs enhancing spiritual resiliency in preparing for disaster recovery efforts in other similar communities.

## Conclusion

This study contributes to a growing literature that supports the role of spirituality and meaning-making in community-led resiliency efforts following disaster. The study demonstrates that spiritually informed resiliency programs and approaches play a role in helping individuals' and communities in the post disaster recovery process and can reinforce post traumatic growth experiences, compassion, and healing. Such strategies and tools foster deeper connections and provide a stronger sense of community engagement and empowerment. Disaster recovery efforts should consider the value of adopting a multi-systemic, collective approach that draws on a shared sense of community identity, engagement, shared norms and beliefs, and spiritual values as essential to promote community resilience and recovery over time. Such resources and values can deepen the community's capacity to take control over their own process of healing, adaptation, and psychosocial restoration and advance for a sustainable recovery process post-disaster. The article signifies that spirituality is an important characteristic of resilience and suggests various tools and strategies for nurturing spiritual resilience among families and communities in post-disaster recovery.

## Data Availability Statement

The original contributions presented in this study are included in the article/supplementary material, further inquiries can be directed to the corresponding author/s.

## Ethics Statement

The studies involving human participants were reviewed and approved by Human Research Ethics Review Board, University of Calgary. The patients/participants provided their written informed consent to participate in this study.

## Author Contributions

JD and CM-H contributed to conception and design of the study. NL, JD, and CM-H organized the database. NL and JD performed the data analysis and wrote the first draft of the manuscript. NL, JD, CM-H, PB-M, and VA wrote sections of the manuscript. All authors contributed to manuscript revision, read, and approved the submitted version.

## Conflict of Interest

The authors declare that the research was conducted in the absence of any commercial or financial relationships that could be construed as a potential conflict of interest.

## Publisher's Note

All claims expressed in this article are solely those of the authors and do not necessarily represent those of their affiliated organizations, or those of the publisher, the editors and the reviewers. Any product that may be evaluated in this article, or claim that may be made by its manufacturer, is not guaranteed or endorsed by the publisher.
